# Experiences of psychiatrists assessing under 18s on an “all age” rota. An evaluation of the current service

**DOI:** 10.1192/bjo.2021.830

**Published:** 2021-06-18

**Authors:** Nicoleta Clarisa Turtoi, Martin Ansell, Trinisha Govender, Helen Bould

**Affiliations:** 1 Gloucestershire Health and Care NHS Foundation Trust; 2 Gloucestershire Health and Care NHS Foundation Trust, Centre for Academic Mental Health, University of Bristol

## Abstract

**Aims:**

Gloucestershire Health Care Trust operates an “all age” out of hours on-call rota, staffed by Registrars and Consultants who cross-cover all psychiatric sub-specialities. Our aim was to understand the challenges faced by psychiatrists of different professional backgrounds when assessing under 18 year olds in a health-based Place of Safety (POS).

**Method:**

We circulated a survey to all psychiatrists on the on-call rota, to gather quantitative and qualitative information on the challenges posed by assessments of under-18s in a POS and assess whether an under-18 specific policy was needed.

**Result:**

Out of the 50 psychiatrists invited, 27 completed this survey (during February 2021). 33.3% of respondents reported that they had completed a POS assessment of an under 18-year-old when a Consultant Child and Adolescent Psychiatrist/Registrar was not available to join. 33.3% of respondents had been asked to complete such an assessment as the sole psychiatrist joining the AMHP, with 24% of those respondents reporting feeling uncomfortable doing these assessments without a second doctor present, and an additional 24% feeling comfortable only sometimes. 48.1% of the doctors surveyed did not invite parents or carers to take part in the assessment, despite this being considered best practice, citing reasons such as: being unaware that this was a possibility, assessments conducted at unsociable hours, safeguarding concerns involving the parents, and social distancing in the context of COVID-19. 41% of respondents had assessed vulnerable young people (children looked after or with a diagnosis of an autism spectrum condition) in the POS and reported that these assessments posed significant challenges to safe discharge planning and identifying appropriate placements. 81.5% felt that a tailored policy for young people was needed. Qualitative findings suggest psychiatrists think such a policy should include clear procedures (flowcharts), potential outcome scenarios/options for safe discharge, referral criteria for CAMHS, contact details for key staff members, a handover protocol. Respondents felt a shared policy with all stakeholders (AMHP service, Mental Health Services, police) was required.

**Conclusion:**

Our survey highlights the challenges for non-CAMHS specialists assessing under 18s in a health-based POS and the need for an under-18 policy to support safe practice. Major themes will be further explored in a focus group to guide policy development.

